# Machine Learning Prediction and Validation of Plasma Concentration–Time Profiles

**DOI:** 10.1021/acs.molpharmaceut.4c01431

**Published:** 2025-05-09

**Authors:** Hiroaki Iwata, Michiharu Kageyama, Koichi Handa

**Affiliations:** † Division of School of Health Science, Department of Biological Regulation, Faculty of Medicine, 13114 Tottori University, 86 Nishi-cho, Yonago 683-8503, Japan; ‡ 26401 Teijin Institute for Bio-medical Research, Teijin Pharma Limited, 4-3-2 Asahigaoka, Hino-shi, Tokyo 191-8512, Japan; § Discovery DMPK Group, Translational Research Department, Axcelead Tokyo West Partners Inc., 4-3-2, Asahigaoka, Hino-shi, Tokyo 191-0065, Japan; ∥ Drug Discovery Chemistry Group, Discovery Science Department, Axcelead Tokyo West Partners Inc., 4-3-2, Asahigaoka, Hino-shi, Tokyo 191-0065, Japan

**Keywords:** population pharmacokinetics, machine learning, remifentanil, virtual data set, real world data set

## Abstract

Recent research has increasingly focused on using machine learning for covariate selection in population pharmacokinetics (PPK) analysis. However, few studies have explored the prediction of plasma concentration profiles of drugs using nonlinear mixed-effect models combined with machine learning. This gap includes limited validation of prediction accuracy and applicability to diverse patient populations and dosing conditions. This study addresses these gaps by using remifentanil as a model drug and applying machine learning models to predict plasma concentration profiles based on virtual and real-world data. We created various training data sets for the virtual data by clustering based on the size and diversity of the test data set. Our results demonstrated high prediction accuracy for virtual and real-world data sets using Random Forest models. These results suggest that machine learning models are effective for large-scale data sets and real-world data with variable dosing times and amounts per patient. Considering the efficiency of machine learning, it offers a fit-for-purpose approach alongside traditional PPK methods, potentially enhancing future pharmacokinetic and pharmacodynamic studies.

## Graphical abstract

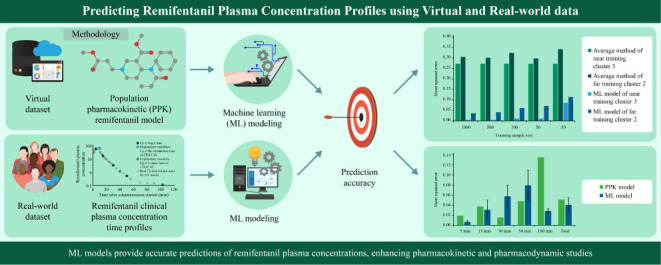

## Introduction

The research and development of new drugs has a significant impact on society due to the persistent unmet medical needs across various diseases.^1^ However, pharmaceutical companies face substantial challenges, with drug development taking over 10 years and costing between $161 million to $4.54 billion (in 2019 US$) per drug.^2^ To address these challenges, model-informed drug discovery and development (MID3) offers a quantitative approach that spans all phases of drug development, from biomarker selection in translational medicine to pharmacoeconomic assessments.^3,4^ In drug development, particularly in pharmacokinetics, rational dose and regimen selection through mathematical models is essential for advancing new drugs to clinical trials.^4^ Population pharmacokinetics (PPK) modeling has been the primary modeling method used.^5^ Building a PPK model involves complex procedures such as data handling, model selection with covariates, and validation.^6^ Based on these difficulties, the increasing availability of clinical data and the growing demand for data science have led to the development and application of various machine learning (ML) methods in clinical pharmacokinetics.^7,8^

The conventional stepwise covariate modeling (SCM) algorithm has been the gold standard for covariate selection. However, SCM has limitations, including issues with removing multiplicity when numerous covariates are involved and is impractical for exploring all possible equations (linear and exponential).^9^ Recent advancements have introduced several ML methods to address these limitations.^10,11^ For example, a study compared classical methods with ML techniques, such as random forest (RF), neural networks (NN), and support vector regression (SVR), applied to NONMEM empirical Bayes estimates. ML methods demonstrated comparable or superior statistical performance compared to SCM and were notably faster in computation, with SCM being 30–100 times slower than NN and SVR, respectively.^11^

Model structure selection in the PPK model also presents challenges. Selecting the appropriate model from a library of existing models requires consideration of factors such as administration routes (e.g., intravenous, oral, or subcutaneous), absorption processes (e.g., first-order, zero-order, or with lag time), elimination mechanisms (e.g., linear or Michaelis–Menten), and compartment numbers. Systematic ML-based selection methods have been developed to improve efficiency in model selection, especially for large data sets or complex models.^12,13^ For instance, a study utilizing genetic algorithms and NN found that ML methods significantly enhance the efficiency of pharmacokinetic model selection and can expedite the initial model selection process, which can then be refined using conventional pharmacometric methods.^12^

Integrating the covariate and model structure selection enables the creation of predictive PPK models. These models are particularly valuable in therapeutic drug monitoring (TDM), where individualized dosing is crucial for drugs with narrow therapeutic windows. In traditional TDM, a patient’s blood sample is collected for drug concentration measurement. This concentration is then used as a sample in PPK modeling to estimate pharmacokinetic parameters using Bayes estimation.^14^ Given the complexities of PPK modeling, ML presents an emerging alternative for TDM. Instead of relying solely on traditional PPK modeling, ML can predict key pharmacokinetic parameters and concentrations from clinical data. Notable examples include studies predicting individual pharmacokinetic parameters for drugs like vancomycin [20–22], cyclosporine [23, 24], and tacrolimus [25–28]. In pharmacokinetic and pharmacodynamic (PK/PD) analysis, the full time–concentration profile of a drug is crucial, as it accounts for subtle temporal changes that are not captured by exposure-response analyses.^15^ Historical PK/PD parameters, such as the ratio of maximum concentration to minimum inhibitory concentration (MIC) and time–concentration above MIC, have been used extensively since the 1980s.^16^ Recent developments include utilizing the complete time–concentration profile for analysis.^17^

In the nonclinical stage, several prediction methods for drug time–concentration profiles in rodents using ML models have recently been published, utilizing chemical structures as descriptors fitted for the drug discovery stages.^18,19^ Surprisingly, before the AI boom in the 1990s and 2000s, prediction methods for time–concentration profiles in human plasma were published for tobramycin^20^ and remifentanil.^21^ Although these studies did not meet modern statistical validation criteria, such as external validation, their predicted accuracy was higher than that of the PPK modeling. Despite their relatively high accuracies, these ML models were not widely accepted; a review article suggested that this might be due to the “black box” nature of predictive inference.^7^ Researchers and clinicians tend to design doses and regimens in easily understandable ways. However, the increasing amount of digitalized clinical data and the development of interpretable ML technology^22^ have changed the landscape. Moreover, considering ML’s much higher processing speed compared to PPK modeling, the current usefulness of ML for predicting concentration–time profiles is evident. Recent publications have demonstrated this for olanzapine,^23^ rosuvastatin,^24^ and valproate.^25^

However, to the best of our knowledge, no study has evaluated ML for predicting plasma concentration–time profiles, particularly regarding the quality and quantity of data sets. In this study, we validated the prediction method for concentration–time profiles using remifentanil, for which clinical data sets of time–concentration profiles and PPK models have been published.^26^ Additionally, since this is the first step of the validation of ML for PPK, the problem set should be as simple as possible. Since remifentanil is an intravenous drug, complexities such as absorption are not an issue in its analysis. Although one previous study attempted to build an ML model for remifentanil concentration–time profiles in human plasma, it was limited by a small number of patients and similar dosing conditions, which restricted its flexibility.^21^ Therefore, we employed two validation methods: first, using the published PPK model, we sampled a large virtual data set for ML validation to investigate sample size limits; second, we used a clinical data set with various dosing regimens to explore dosing limits.^26^

## Materials and Methods

### Overall Workflow

In this study, we prepared virtual and real-world data sets of remifentanil and built ML models using these data sets. Figure 1 illustrates this workflow.

**Figure 1:**
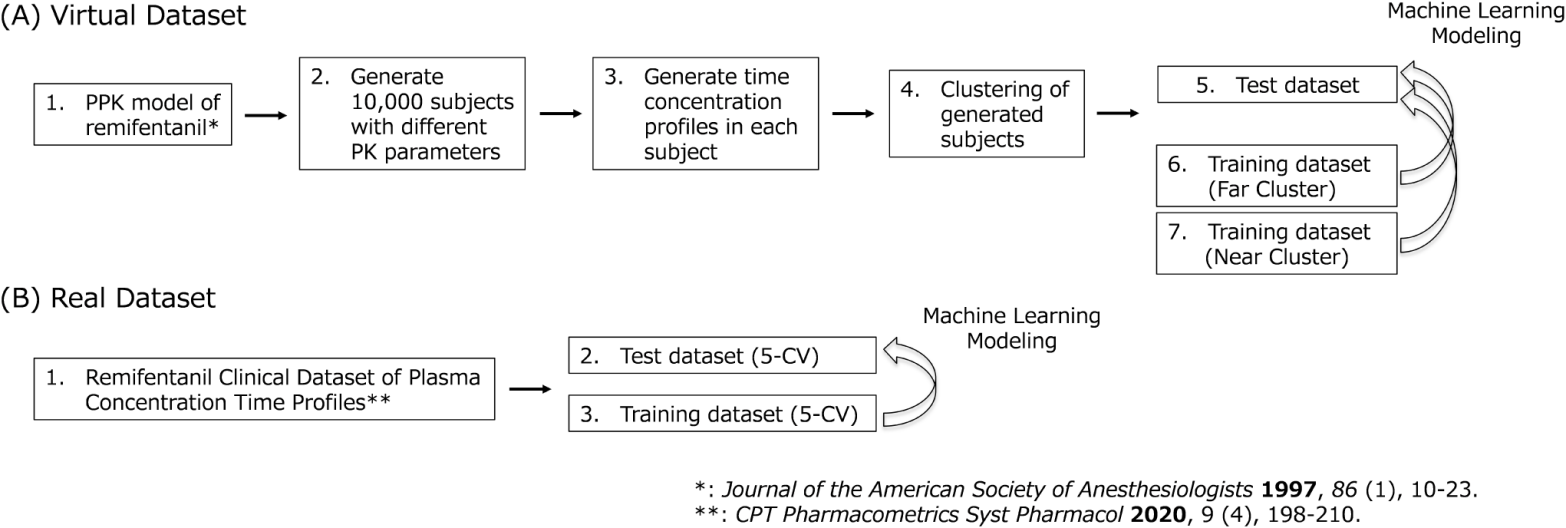
Overall workflow of the validation study.

Virtual data set (A) and real-world data set (B) were validated using different processes.

### Data Set

#### Virtual Data Set

##### Generation of Data Set

A large virtual data set was prepared from samples using published equations from a PPK study (Figure 1A-1).^26^ Sampling followed the following equations:

1 $$\begin{array}{l} V1=\left(5.1-0.0201\times \left(\mathrm{Age}-40\right)+0.072\times \left(\mathrm{LBM}-55\right)\right)\times \mathrm{Exp}\left(\eta_{V1}\right)\\ \eta_{V1}∼N\left(0,{\omega_{V1}}^{2}\right),\;{\omega_{V1}}^{2}=0.26 \end{array}$$

2 $$\begin{array}{l} V2=\left(9.82-0.0811\times \left(\mathrm{Age}-40\right)+0.108\times \left(\mathrm{LBM}-55\right)\right)\times \mathrm{Exp}\left(\eta_{V2}\right)\\ \eta_{V2}∼N\left(0,{\omega_{V2}}^{2}\right),\;{\omega_{V2}}^{2}=0.29 \end{array}$$

3 $$\begin{array}{l} V3=5.42\times \mathrm{Exp}\left(\eta_{V3}\right)\\ \eta_{V3}∼N\left(0,{\omega_{V3}}^{2}\right),\;{\omega_{V3}}^{2}=0.66 \end{array}$$

4 $$\begin{array}{l} \mathrm{CL}1=\left(2.6-0.0162\times \left(\mathrm{Age}-40\right)+0.0191\times \left(\mathrm{LBM}-55\right)\right)\times \mathrm{Exp}\left(\eta_{CL1}\right)\\ \eta_{CL1}∼N\left(0,{\omega_{\mathrm{CL}1}}^{2}\right),\;{\omega_{\mathrm{CL}1}}^{2}=0.14 \end{array}$$

5 $$\begin{array}{l} \mathrm{CL}2=\left(2.05-0.0301\times \left(\mathrm{Age}-40\right)\right)\times \mathrm{Exp}\left(\eta_{CL2}\right)\\ \eta_{CL2}∼N\left(0,{\omega_{CL2}}^{2}\right),\;{\omega_{CL2}}^{2}=0.36 \end{array}$$

6 $$\begin{array}{l} \mathrm{CL}3=\left(0.076-0.00113\times \left(\mathrm{Age}-40\right)\right)\times \mathrm{Exp}\left(\eta_{CL3}\right)\\ \eta_{CL3}∼N\left(0,{\omega_{\mathrm{CL}3}}^{2}\right),\;{\omega_{\mathrm{CL}3}}^{2}=0.41 \end{array}$$

7 Age∼U(20,85)

8 LeanBodyMass(LBM)∼U(36,76)

The virtual data set comprised 10,000 PK sets (virtual subjects) (Figure 1A-2). These PK parameters were used to simulate remifentanil plasma concentration (Cp)-time profiles using a 3-compartment model. For this virtual data set, we set the dosing rate and infusion time as constants at 227 μg/min/body and 10 min, respectively, based on observed clinical settings (Figure 1A-3). Sampling and simulations were performed by using the RxODE library in R4.3.3.

##### Virtual Subjects Splitting into Test and Training Sets by Clustering

To evaluate model dependency on the data set, we created two different training data sets against one test data set through a clustering process (Figure 1A-4). First, 10,000 virtual subjects were divided into 10 clusters using hierarchical clustering based on age, lean body mass (LBM), Cp at 10 min, and Cp at 40 min using the agglomerative clustering function in scikit-learn 1.3.0 in Python 3. These properties were chosen because age and LBM are significant covariates in PPK equations, and Cp at 10 and 40 min reflects the infusion’s terminal state and the elimination phase of remifentanil, respectively.

Next, we randomly selected a test data set, named Cluster 1 (Figure 1A-5), and identified the training data set furthest from Cluster 1, named Cluster 2 (far cluster) (Figure 1A-6). The training data set nearest to Cluster 1 was named Cluster 3 (near cluster) (Figure 1A-7).

##### Number of Subjects in the Training Data Set

Since clinical data sets can be of variable sizes, it is important to understand how the model works under different data set sizes. Consequently, we investigated model dependency on the size of the training data sets. For each training data set, we selected 1,000, 500, 100, 50, and 10 subjects. For the test data set, we randomly selected 100 subjects from Cluster 1.

### Real-World Data Set

The real-world data set was extracted from a previous report, selecting the time, Cp, age, and LBM of 64 clinical subjects.^26,27^ Since remifentanil is known to be rapidly metabolized by esterases,^28^ acetonitrile was immediately added to the blood samples during collection to deactivate esterase activity.^26^ One subject with poor Cp time points (ID No. 11) was removed from the original 65 subjects (Figure 1B-1). The statistical properties of the participants are summarized in Table 1.

**Table 1:** Statistical Properties of the Subjects

	Dose	Rate	Age	LBM	TINFCAT
Metrics	μg	μg/min/body	Years	Kg	Min
Average	2431	227	47	55	10
Standard deviation	2296	101	21	10	4
Maximum	15,000	750	85	76	20
Median	1863	216	41	57	10
Minimum	675	63	20	36	4

#### Details of Data Set

Each subject had varying time points for Cp, with some having fewer samples and others having more samples in the clinical stage. This setting of sampling time would be determined by the fixed exact time or some-fold of the length of infusion time. To ensure a fair analysis, we extracted time points to create two data sets. The first data set included subject properties and Cp at fixed nearest time points (5, 15, 30, 50, and 100 min), the termination time of infusion (TINFCAT), and four times the TINFCAT. The second data set included subject properties and Cp at 0.5, 1.5, 3, 5, and 10 times the TINFCAT, the termination time of TINFCAT, and four times the TINFCAT. Finally, the data set was divided into test and training sets using 5-fold cross-validation (CV) (Figure 1B-2, 3).

### Proposed Machine Learning Method

In this study, we implemented eight different ML methods: least absolute shrinkage and selection operator, ridge regression, k-nearest neighbors, support vector machine, RF, gradient boosting (GB), extreme gradient boosting (XGB), and neural network using Scikit-learn 1.3.0 in Python 3. We set 20% of the training data as validation data and conducted parameter tuning for each ML method through a grid search. Performance evaluation was then carried out using optimal parameters. The list of the eight ML methods and the parameter ranges used in the grid search are provided in Table S1. The optimal parameters were selected based on performance metrics. The training and test data sets are listed in Table 2. We used seven explanatory variables: target time, infusion rate, age, LBM, TINFCAT, and Cp at the termination time of TINFCAT, and Cp four times after TINFCAT.

**Table 2:** Conditions for Each Machine Learning Model

Objective Variables	Explanatory Variables	Data Set Origin	Target Time	Test Data Set	Training Data Set	Number of Training Subjects
Cp at target time	Target time, infusion rate, Age, LBM, TINFCAT, Cp at the termination time of TINFCAT, Cp at 4-times later of TINFCAT	Virtual	5, 15, 30, 50, 100 min	Cluster 1	Cluster 2 (far cluster)	1000
						500
						100
						50
						10
					Cluster 3 (near cluster)	1000
						500
						100
						50
						10
		Real-world	5, 15, 30, 50, 100 min	5-fold CV		
			0.5, 1.5, 3, 5, 10 times (×) of TINFCAT	5-fold CV		

For the Target Time, we assumed several sampling time points that could be obtained in early clinical stages, such as Phase I studies. For Cp at the termination time of TINFCAT and Cp four times later, we assumed sampling points obtainable in later clinical stages, such as Phase II or III, where PPK studies are generally performed. The prediction workflow, considering the above description, is shown in Figure 2.

**Figure 2:**
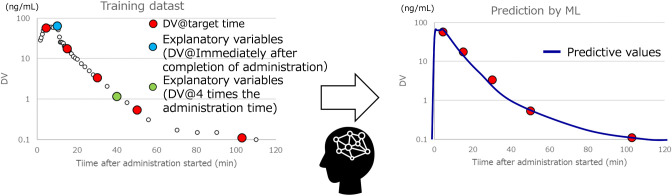
Prediction workflow for this study.

This figure shows prediction workflow using the proposed ML method with a real data set. The ML model was built using the training data set, and remifentanil plasma concentration–time profiles were predicted based on specified times and other variables. The explanatory variables included target time, infusion rate, age, LBM, TINFCAT, Cp at the termination time of TINFCAT, and Cp four times later than that of TINFCAT. Consequently, instead of the target time, any specified time can be input to predict Cp at that time.

### Compared Methods

#### Average Method

To assess the effectiveness of the proposed ML method using a virtual data set, we employed the average method as a control method. This method is commonly used in ML research.^18,29^ Additionally, this method is consistent with the Naïve Average Data approach, which is one of the classical PPK modeling^30^ used when the sampling time points and doses are the same in patients. Predicted values were calculated based on the average values at specific time points in the training data set. Although this method does not account for individual differences in Cp, it is computationally efficient and suitable for large virtual data sets.

### Population Pharmacokinetics Model

Constructing a PPK model for a large virtual data set is challenging due to the sample size. However, for smaller, real-world data sets, PPK modeling is feasible. We compared the proposed ML model with the PPK model by using real-world data. To build the PPK model, we included all Cp data at the termination time of TINFCAT and Cp four times later without separation of the training and test. We used TINFCAT and Cp only four times later, excluding Cp at other time points that were to be predicted. This approach was taken to prevent information leakage, which could occur if the predicted value were included in the PPK analysis for modeling when comparing ML model predictions for the test data set.^31^

A robust PPK model requires sufficient subjects; a small sample size can lead to poor model performance.^32^ The PK structure and covariates were based on a 3-compartment model with a multiplicative error model, following a previous report.^26^ Stepwise methods were used to determine the equations and coefficients for the clearance and volume of distribution. The parameters and equations used are listed in Table S2.

### Metrics

To evaluate and compare the models, we calculated the *R* ^2^-value and mean squared error (MSE) using the following equations:

9 $$\mathrm{MSE}=\frac{1}{n}\overset{n}{\underset{i=1}{\sum }}\left(\log\left(\overset{®}{y_{i}\;}\right)-\log(y_{i}\right){(}^{2}$$

10 $$R^{2}‐\mathrm{value}=1-\frac{SS_{res}}{SS_{tot}}$$

where SS_res_ is the sum of squared residuals, and SS_tot_ is the total sum of squares. These calculations were performed manually using Microsoft Excel (Office 365 version).

## Results and Discussion

### Performance Evaluation Using Virtual Data Set

#### Dependency on the Training Data Set’s Distance and Size Against the Test Set

To assess the dependency of the proposed ML model on the diversity and size of the training data set, various training data sets were prepared from a generated virtual data set. We implemented eight ML models. The results of the clustering are listed in Table S3, showing that each cluster has over 500 virtual subjects. Cluster 1 was assigned as the test cluster, whereas Clusters 2 and 3 were assigned as the furthest (distance: 56.52) and nearest (distance: 20.05) training clusters, respectively. Random sampling from Clusters 2 and 3 was performed to prepare training data sets of different sizes, including 1,000, 500, 100, 50, and 10 virtual subjects. Tables S4 and S5 list the MSE and *R* ^2^ values for each setting.

The predictivity (MSE) of each ML model against 100 test data sets randomly selected from Cluster 1 is shown in Figure 3AB. Here, we discuss the three ML models with the highest accuracy: RF, GB, and XGB. The results indicate that the predictivity using the near training cluster was superior to that of the far training cluster, regardless of the training size (average MSE and *R* ^2^ values: RF: 0.0225 and 0.9895, GB: 0.0173 and 0.9919, and XGB: 0.0368 and 0.9828 for the near cluster; RF: 0.0645 and 0.9699, GB: 0.0599 and 0.9721, and XGB: 0.0695 and 0.9675 for the far cluster). Both ML models outperformed the average method (average MSE and *R* ^2^ values: 0.2739 and 0.8721 for the near cluster; 0.3164 and 0.8523 for the far cluster).

**Figure 3:**
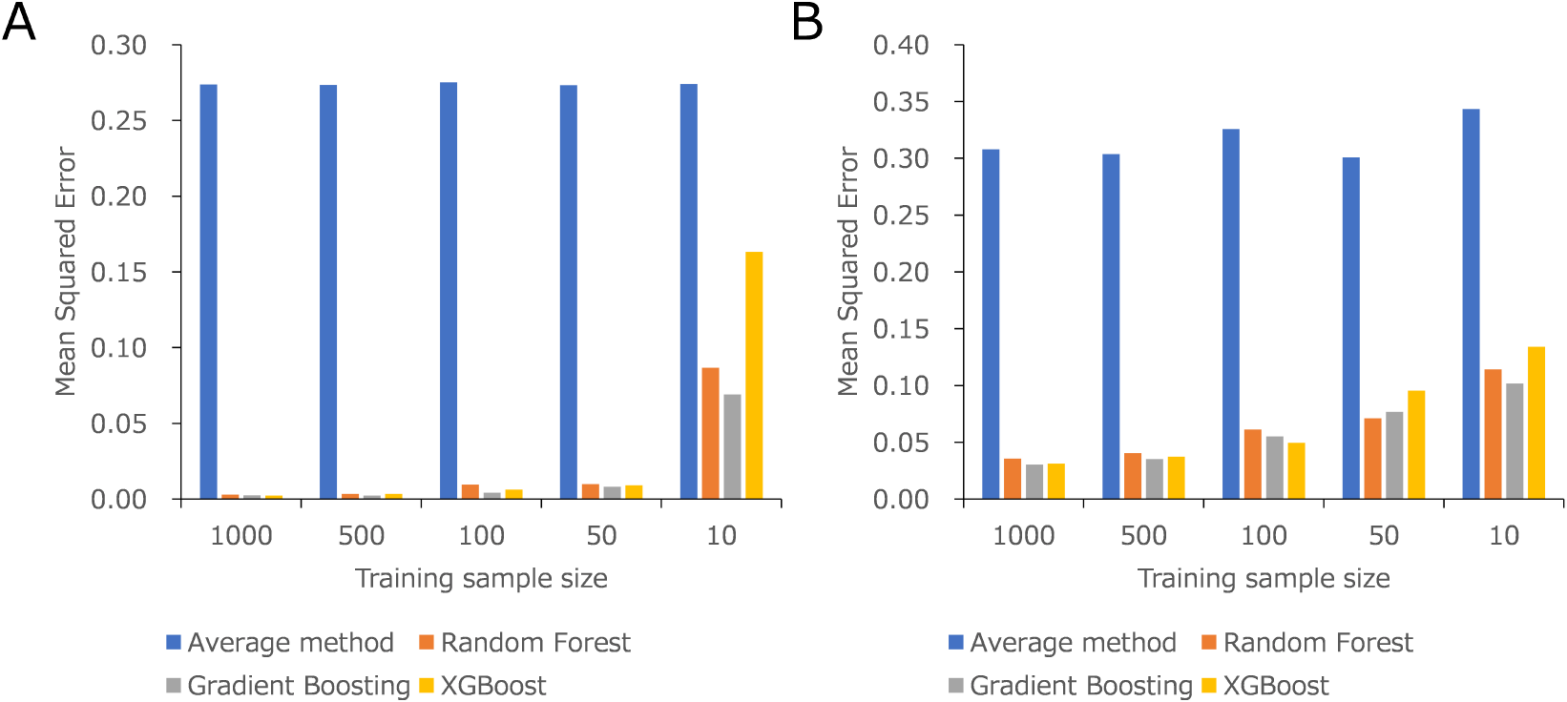
Dependency on the distance and size of the training data set of predictivity in the proposed ML model in the virtual data set using near (A) and far (B) training data set.

Focusing on the MSE for the near cluster, the MSE for 10 training subjects was significantly higher (MSE: RF: 0.0867, GB: 0.0691, and XGB: 0.1632) than for other settings (MSE: RF: 0.0099 for 50, 0.0096 for 100, 0.0033 for 500, and 0.0029 for 1000 training subjects, GB: 0.0082, 0.0043, 0.0023, and 0.0025, and XGB: 0.0090, 0.0062, 0.0034, and 0.0023). This indicates that more than 50 subjects are sufficient for precise predictions when using a similar training data set to the test data set. For the far cluster, the MSE for 10 training subjects was also much higher (MSE: RF: 0.1143, GB: 0.1017, and XGB: 0.1340). However, the MSE decreased linearly with an increasing number of training subjects (MSE: 0.0711 for 50, 0.0612 for 100, 0.0404 for 500, and 0.0357 for 1000 training subjects; GB: 0.0767, 0.0551, 0.0353, and 0.0305; and XGB: 0.0954, 0.0495, 0.0373, and 0.0312). This trend indicates that even when the training subjects are far from the test data, a larger training size (over 100 subjects) can still contribute to building an effective predictive ML model.

The MSE was compared according to the size of the training data set (from 1000 to 10) against the test data set.

### Dependency on the Target Time Points

To understand the features of the proposed ML models, we analyzed the differences in predictivity at various time points after administration. Based on previous results, ML models using 100 virtual subjects were compared with the average method (Figure S1). The MSE and *R* ^2^-values for each setting are listed in Tables S6 and S7, respectively. Figure 4A,B show the MSE values for near and far training clusters, respectively. The MSE and *R* ^2^ values for the overall target time points using the near cluster were better (RF: 0.0096 and 0.9955, GB: 0.0043 and 0.9980, and XGB: 0.0062 and 0.9971, respectively) than those using the far clusters (RF: 0.0612 and 0.9714, GB: 0.0551 and 0.9743, and XGB: 0.0495 and 0.9769, respectively). Near and far training clusters had much higher predictivity than those of the average method (0.2752 and 0.8715 for near clusters and 0.3259 and 0.8478 for far clusters, respectively).

**Figure 4:**
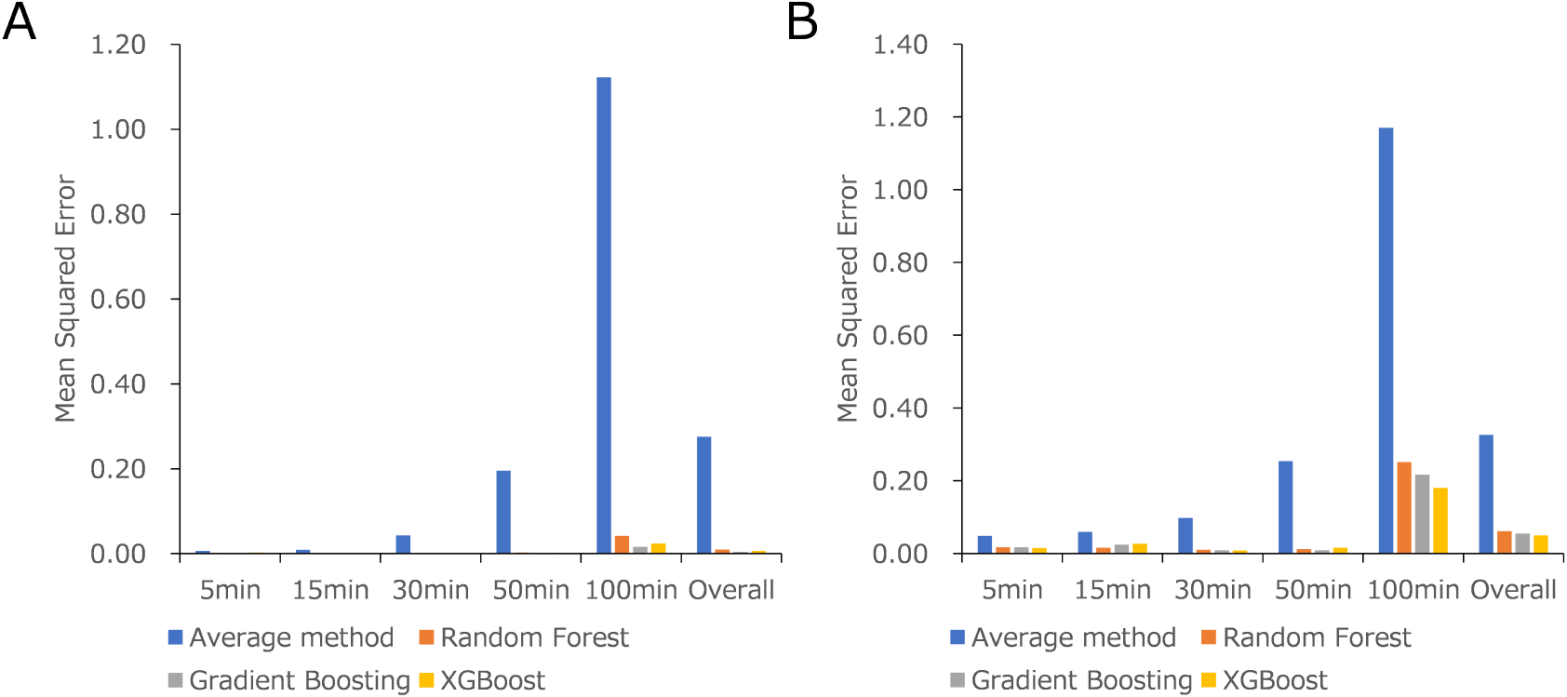
MSE of proposed ML models on each time point when using near (A) and far (B) training clusters, with a training size of 100.

Focusing on the MSE, it is observed that the MSE at late time points (50–100 min: RF: 0.0023–0.0418, GB: 0.0013–0.0164, and XGB: 0.0020–0.0240 for the near training model and RF: 0.0116–0.2512, GB: 0.0089–0.2162, and XGB: 0.0163–0.1804 for the far training model) was higher than at early time points (5–30 min: RF: 0.0011–0.0017, GB: 0.0010–0.0015, and XGB: 0.0014–0.0025 for the near training model and RF: 0.0102–0.0172, GB: 0.0087–0.0243, and XGB: 0.085–0.0258 for the far training model). The Cp values were also analyzed, leading to a similar conclusion that predictivity was better at higher Cp values. This indicates that predictivity worsens over time, consistent with the increased difficulty in predicting the terminal phase due to complex elimination processes.^18,19^ However, even at 100 min, the MSE of the near and far training clusters of the ML model was significantly lower than those of the average model.

The MSE was compared according to the sampling time points (from 5 to 100 min) when using 100 training samples from the near and far data sets against the test data set.

### Performance Evaluation Using Real-World Data Set

#### Predictivity of Real Plasma Concentration–Time Profiles at Exact Target Time Points

In addition to validation with the virtual data set, we developed an ML model using the remifentanil real-world data set under a 5-fold CV and compared its performance with that of the PPK model built in this study. We implemented three ML models: RF, GB, and XGB. However, we focused our discussion on RF, as it showed relatively high accuracy with the virtual data set. Results for the other ML methods are provided in the Supporting Information. The MSE and *R* ^2^-values for each setting are listed in Tables S8 and S9, respectively. Notably, the overall MSE for the RF model (0.0405 ± 0.0132) was slightly lower than for the PPK model (0.0508), as shown in Figure 5A. Similarly, the *R* ^2^-values were 0.9573 for the PPK model and 0.9606 ± 0.0117 for the RF model.

**Figure 5:**
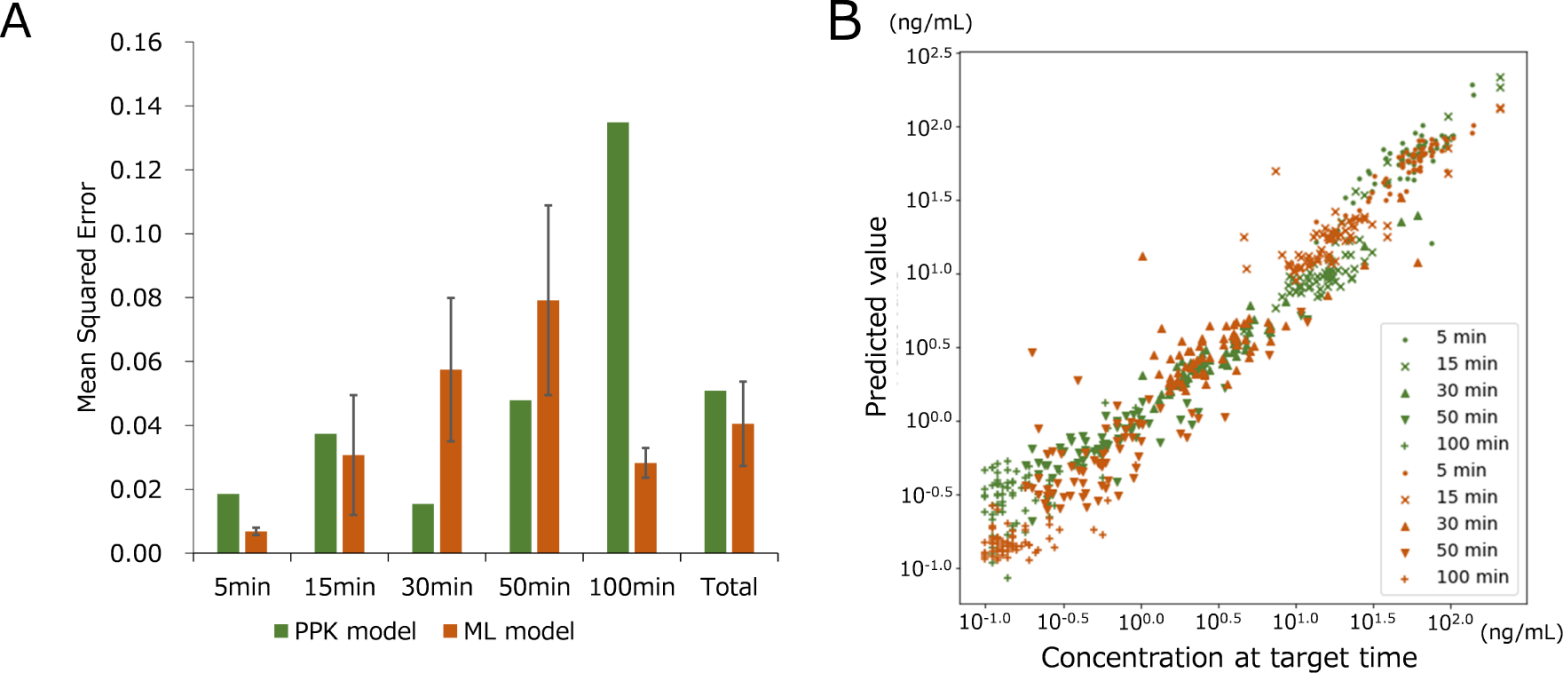
MSE (A) and plot (B) of proposed ML and PPK models at each target time point on the basis of the exact time.

Focusing on MSE at specific time points, at 5 and 15 min, the MSEs for the PPK model (0.0186 and 0.0374) were higher than for the RF model (0.0068 ± 0.0012 and 0.0307 ± 0.0188). Conversely, at 30 and 50 min, the MSEs for the PPK model (0.0154 and 0.0479) were lower than for the RF model (0.0574 ± 0.0224 and 0.0792 ± 0.0297). At 100 min, the MSE for the PPK model (0.1349) was significantly higher than for the RF model (0.0283 ± 0.0046). Figure 5B shows a plot of log-transformed predicted versus observed values for both models. Visually, the RF model predicted better at lower concentrations (−1.0 to – 0.5); however, in the middle range (−0.5 to 1.0), the RF model’s predictions had a larger spread. These results suggest that the RF model performs better at earlier and later time points with lower plasma concentrations, whereas the PPK model performs better at intermediate time points with higher plasma concentrations.

(A) The MSE was compared according to the sampling time points (from 5 to 100 min) in the clinical data set. (B) Scattered plot whose data is from (A: PPK in green, and ML in orange); the concentration observed (*x*-axis) and predicted (*y*-axis) values are transformed into log-values.

### Predictivity of Real Plasma Concentration–Time Profiles on Infusion Time

We also compared the predictivity of the RF and PPK models based on infusion time, which varied for each subject (Table 1 and Figure 6). The MSE and *R* ^2^-values for each setting are shown in Tables S10 and S11. Overall, the RF model’s performance (MSE: 0.0422 ± 0.0113, *R* ^2^: 0.9569 ± 0.0105) was equivalent to that of the PPK model (MSE: 0.0491, *R* ^2^: 0.9531). During infusion, both models demonstrated a high predictivity. Immediately after infusion (TINFCAT × 1.5), the RF (MSE: 0.0308 ± 0.0078) was lower than the PPK model (MSE: 0.0641). At longer times after infusion (TINFCAT × 3 and × 5), the MSEs of the PPK model (0.0149 and 0.0157) were higher than the RF model’s (0.0573 ± 0.0180 and 0.0425 ± 0.0166). At a much longer time after infusion (TINFCAT × 10), the MSE of the PPK model (0.1428) was significantly higher than that of the RF model (0.0696 ± 0.0177). These findings are consistent with those in the previous section: the RF model performs well at earlier and later time points with lower plasma concentrations, whereas the PPK model performs well at intermediate time points with higher plasma concentrations.

**Figure 6:**
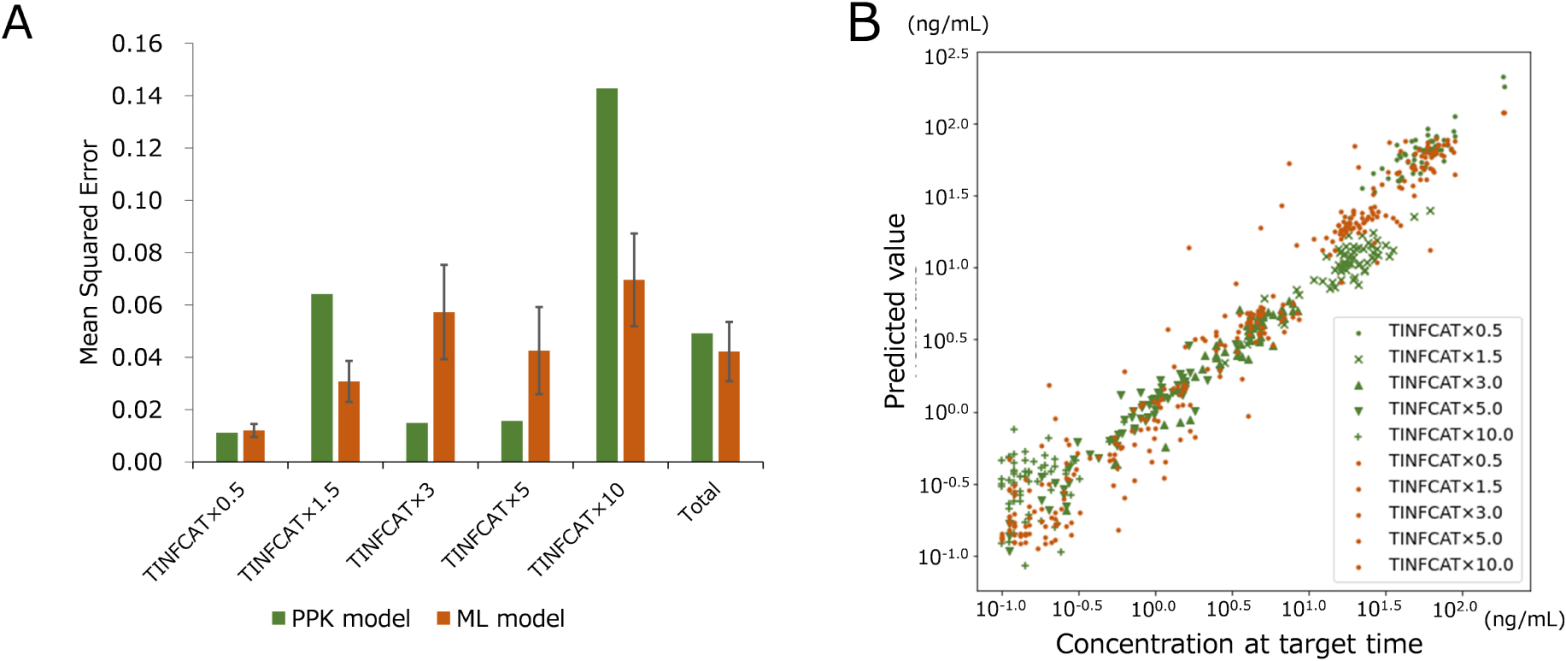
MSE (A) and plot (B) of proposed ML and PPK models at each time point based on the infusion time.

In conclusion, based on these predictive results (Figures 5 and 6), the RF model proposed in this study shows high predictivity for plasma concentration. This indicates a new era of prediction methods for clinical plasma drug concentrations, with RF offering faster analysis compared to PPK.^11^ Despite this, the PPK model’s predictivity remains robust even when built using a small data set (e.g., at the termination time of TINFCAT, Cp four times later than TINFCAT) to maintain a sufficient number of subjects. The time–concentration profile of remifentanil is relatively uncomplicated, partially due to its administration by infusion, which bypasses the absorption process. Predicting concentration–time profiles becomes more challenging with drugs involving initial absorption processes and factors such as first-pass liver metabolism.^33^ However, this study has some limitations. First, the data set used is relatively small, which might affect the generalizability of the findings. Second, the study focuses on remifentanil, a drug with a straightforward pharmacokinetic profile, limiting the applicability of the results to drugs with more complex profiles. Additionally, the RF model’s performance was evaluated under specific conditions, and its predictive accuracy may vary depending on different data characteristics or clinical settings. Therefore, to conclusively determine the superior method, further research should address drugs with more complex pharmacokinetics, such as those with double peaks.^34^ Future studies should also consider larger and more diverse data sets to enhance the robustness and generalizability of the findings.

(A) The MSE was compared according to the sampling time points (from TIFCAT × 0.5 to TIFCAT × 10) in the clinical data set. (B) Scattered plot whose data is from (A: PPK in green, and ML in orange); the concentration observed (*x*-axis) and predicted (*y*-axis) values are transformed into log-values.

## Conclusion

This study utilized remifentanil as a model to evaluate the effectiveness of ML models in predicting plasma concentration profiles, validated using virtual and real data sets. Our findings indicate that the ML model achieved higher prediction accuracy with nearby training clusters and larger training sample sizes. Even with distant training clusters, the accuracy remained acceptable with increased training samples. When applied to real data, the ML model demonstrated prediction accuracy equal to or surpassing that of the PPK model, indicating its robustness for individual patient PK predictions. Therefore, ML models are suitable for large-scale data sets and real-world data with variable dosing times and amounts. Given the speed and efficiency of ML, it offers a significant practical advantage over traditional PPK models, particularly for extensive data sets. However, the difference in prediction accuracies should be further investigated, as this study did not include a sufficient number of real samples for PPK modeling. Future studies should further validate this technique by applying it to compounds with more complex PK profiles, such as those with oral administration or double plasma peaks. This research marks a potential shift toward more rapid and accurate PK predictions using ML, tailored to specific analytical needs.

## Supplementary Material





## Data Availability

Data is provided in Supplementary_Data_File.xlsx.

## Notes

Table S1: the list of 8 machine learning methods and grid search range; Table S2: PPK model built in this study using a real-world data set; Table S3: cluster sizes and distances of virtual data sets; Table S4: MSE of the average method and the proposed ML model using virtual data sets; Table S5: *R* ^2^ values for the average method and proposed ML model using the virtual data sets; Table S6: MSE of the average method and proposed ML model using virtual data sets of size 100; Table S7: *R* ^2^ values of the average method and proposed ML model using virtual data sets with a sample size of 100; Table S8: MSE of PPK and proposed ML model using real data sets for each specified time point and overall; Table S9: *R* ^2^ values of PPK and proposed ML models using real data sets for each specified time point and overall; Table S10: MSE of PPK and proposed ML models using real data sets for each fold of time points during the infusion period and overall; Table S11: *R* ^2^ value of PPK and proposed ML models using real data sets for each fold of time points during the infusion period and overall; Figure S1: comparison of predictivity at different Cp values using 100 training subjects from the near (A) and far (B) clusters (PDF)
(XLSX)

## Abbreviations

PPK: population pharmacokinetics
PK/PD: pharmacokinetic and pharmacodynamic
MID3: model-informed drug discovery and development
ML: machine learning
SCM: stepwise covariate modeling
RF: random forest
NN: neural networks
SVR: support vector regression
TDM: therapeutic drug monitoring
MIC: minimum inhibitory concentratio
TINFCAT: termination time of infusion
CV: cross-validation
LBM: lean body mass
Cp: remifentanil plasma concentration
MSE: mean squared error
GB: gradient boosting
XGB: extreme gradient boosting
