# Supplementary material for: Machine Learning Prediction and Validation of Plasma Concentration–Time Profiles
Source: Mol Pharm. 2025 May 9;22(6):2976–84. doi: 10.1021/acs.molpharmaceut.4c01431 (PMC12135056; doi:10.1021/acs.molpharmaceut.4c01431)
Supplement: Supplementary file 1 [file mp4c01431_si_001.pdf]

SUPPLEMENTARY DATA

Machine Learning Prediction and Validation of Plasma Concentration Time Profiles

Hiroaki Iwata[1*], Michiharu Kageyama[2,3], and Koichi Handa[2,4*]

[1] Division of School of Health Science, Department of Biological Regulation, Faculty of Medicine, Tottori University, 86 Nishi-cho, Yonago 683-8503, Japan
[2] Teijin Institute for Biomedical Research, Teijin Pharma Limited, 4-3-2 Asahigaoka, Hino-shi, Tokyo 191-8512, Japan.
[3] Discovery DMPK Group, Translational Research Department, Axcelead Toyko West Partners Inc., 4-3-2, Asahigaoka, Hino-shi, Tokyo, 191-0065, Japan
[4] Drug Discovery Chemistry Group, Discovery Science Department, Axcelead Toyko West Partners Inc., 4-3-2, Asahigaoka, Hino-shi, Tokyo, 191-0065, Japan

[*]Corresponding author: Hiroaki Iwata, Division of School of Health Science, Department of Biological Regulation, Faculty of Medicine, Tottori University, 86 Nishi-cho, Yonago 683-8503, Japan
Tel: +81- 859-38-7615
E-mail: iwata.hiroaki@tottori-u.ac.jp

[*]Co-corresponding author: Koichi Handa, Drug Discovery Chemistry Group, Discovery Science Department, Axcelead Toyko West Partners Inc., 4-3-2 Asahigaoka, Hino-shi, Tokyo, 191-0065, Japan
Tel: +81- 42-586-8260
E-mail: koichi.handa@axcelead-twp.com

Table S1: The list of 8 machine learning methods and grid search range

Methods	Grid search range
LASSO	'alpha': [0.1, 0.5, 1, 5, 10]
Ridge	'alpha': [0.1, 1, 10, 100]
KNN	'n_neighbors': [3, 5, 7, 9]<br>'weights': ['uniform', 'distance']<br>'p': [1, 2] # 1: Manhattan, 2: Euclidean
SVM	'C': [0.01, 0.1, 1]<br>'kernel': ['linear', 'rbf', 'poly']<br>'gamma': ['scale', 'auto']
Random Forest	'n_estimators': [100, 200, 300]<br>'max_depth': [None, 5, 10]
Gradient Boosting	'n_estimators': [100, 200, 300]<br>'learning_rate': [0.01, 0.1, 0.2]<br>'max_depth': [3, 5, 7]
XGBoost	'n_estimators': [100, 200, 300]<br>'learning_rate': [0.01, 0.1, 0.2]<br>'max_depth': [3, 5, 7]
Neural Network	'hidden_layer_sizes': [(50,), (100,), (50, 50), (100, 100)]<br>'activation': ['relu', 'tanh']<br>'solver': ['adam', 'sgd']<br>'alpha': [0.0001, 0.001, 0.01]<br>'learning_rate': ['constant', 'adaptive']<br>'max_iter': [200, 400, 800]

Table S2: PPK Model Built in This Study using a Real-World Dataset

This was performed in Phoenix64 NLME (version 8.3.4.295).

$V1 = \theta_{V1} \times EXP(\eta_{v1})$

$\eta_{v1} \sim N(0, \omega_{v1}^2)$

$V2 = \theta_{V2} \times EXP(\eta_{v2})$

$\eta_{v2} \sim N(0, \omega_{v2}^2)$

$V3 = \theta_{V3} \times EXP(\eta_{v3})$

$\eta_{v3} \sim N(0, \omega_{v3}^2)$

$CL1 = \theta_{CL1} \times (AGE/mean(AGE))^{dCL1dAGE} \times (LBM/mean(LBM))^{dCL1dLBM} \times EXP(\eta_{CL1})$

$\eta_{CL1} \sim N(0, \omega_{CL1}^2)$

$CL2 = \theta_{CL2} \times EXP(\eta_{CL2})$

$\eta_{CL2} \sim N(0, \omega_{CL2}^2)$

$CL3 = \theta_{CL3} \times (LBM/mean(LBM))^{dCL3dLBM} \times EXP(\eta_{CL3})$

$\eta_{CL3} \sim N(0, \omega_{CL3}^2)$

Parameter	Units	Estimate	%RSE	%IIV	%RSE	Shrinkage
θ_{V1}	L	1.7	10.8	1.2	5.0	1.0
θ_{V2}	L	8.9	5.5	2.4	6.1	0.9
θ_{V3}	L	20.4	12.1	128.4	21.9	0.5
θ_{CL1}	L/min	2.4	2.2	2.6	13.0	0.8
θ_{CL2}	L/min	1.3	10.6	26.7	34.0	0.6
θ_{CL3}	L/min	0.2	6.3	0.4	7.3	1.0
dCldAGE	-	-0.3	-10.5	-	-	-
dCl3dLBM	-	0.0	3.0	-	-	-
dCldLBM	-	0.2	17.8	-	-	-
stdev0	-	0.2	9.4	-	-	-

Table S3: Cluster Sizes and Distances of Virtual Datasets

Distance between each cluster	Cluster 1 (Test Cluster)	Cluster 2 (Far Cluster)	Cluster 3 (Near Cluster)	Cluster 4	Cluster 5	Cluster 6	Cluster 7	Cluster 8	Cluster 9	Cluster 10	**Number of samples**
Cluster 1 (Test Cluster)	0	56.52	20.05	20.15	36.43	31.66	38.35	54.43	42.59	24.36	**1,005**
Cluster 2	56.52	0	40.2	39.78	20.34	37.34	26.75	18.53	20.72	49.62	**1,681**
Cluster 3	20.05	40.2	0	15.64	20.86	28.19	19.16	35.25	30.52	29.72	**1,072**
Cluster 4	20.15	39.78	15.64	0	20.04	13.53	28.88	41.93	23.09	14.9	**1,024**
Cluster 5	36.43	20.34	20.86	20.04	0	22.09	16.05	23.43	12.63	32.12	**842**
Cluster 6	31.66	37.34	28.19	13.53	22.09	0	36.2	45.3	17.29	13.23	**705**
Cluster 7	38.35	26.75	19.16	28.88	16.05	36.2	0	16.63	28.24	43.34	**931**
Cluster 8	54.43	18.53	35.25	41.93	23.43	45.3	16.63	0	31.49	55.31	**1,184**
Cluster 9	42.59	20.72	30.52	23.09	12.63	17.29	28.24	31.49	0	30.21	**967**
Cluster 10	24.36	49.62	29.72	14.9	32.12	13.23	43.34	55.31	30.21	0	**589**

Table S4: MSE of the Average Method and the Proposed ML Model Using Virtual Datasets

Training Cluster	Predictive models	Number of Training Subjects					
		1000	500	100	50	10	Mean
Near Training Cluster 3	Average Method	0.2737	0.2734	0.2752	0.2732	0.2740	0.2739
	LASSO	0.1770	0.1762	0.1800	0.1778	0.2281	0.1878
	Ridge	0.1839	0.1830	0.1799	0.1843	0.3581	0.2178
	KNN	0.1253	0.1180	0.1725	0.2046	0.3728	0.1986
	SVM	0.1046	0.1393	0.2050	0.2013	0.2676	0.1836
	Random Forest	0.0029	0.0033	0.0096	0.0099	0.0867	0.0225
	Gradient Boosting	0.0025	0.0023	0.0043	0.0082	0.0691	0.0173
	XGBoost	0.0023	0.0034	0.0062	0.0090	0.1632	0.0368
	Neural Network	0.0333	0.0406	0.0886	0.1298	0.4296	0.1444
Far Training Cluster 2	Average Method	0.3079	0.3038	0.3259	0.3008	0.3435	0.3164
	LASSO	0.1889	0.1906	0.1765	0.1892	0.9601	0.3411
	Ridge	0.2240	0.2280	0.2127	0.2137	0.8479	0.3453
	KNN	0.1787	0.1998	0.2485	0.1854	0.3538	0.2332
	SVM	0.1432	0.2002	0.1896	0.1942	0.2290	0.1912
	Random Forest	0.0357	0.0404	0.0612	0.0711	0.1143	0.0645
	Gradient Boosting	0.0305	0.0353	0.0551	0.0767	0.1017	0.0599
	XGBoost	0.0312	0.0373	0.0495	0.0954	0.1340	0.0695
	Neural Network	0.6238	0.1322	1.1472	0.9372	1.8509	0.9383

Table S5: R^2 values for the Average method and Proposed ML model using the Virtual Datasets

Predictive Model	Training Cluster	Number of Training Subjects					
		1000	500	100	50	10	Mean
Near Training Cluster 3	Average Method	0.8722	0.8723	0.8715	0.8724	0.8720	0.8721
	LASSO	0.9174	0.9177	0.9159	0.9170	0.8935	0.9123
	Ridge	0.9141	0.9145	0.9160	0.9139	0.8328	0.8983
	KNN	0.9415	0.9449	0.9195	0.9045	0.8259	0.9073
	SVM	0.9512	0.9350	0.9043	0.9060	0.8750	0.9143
	Random Forest	0.9986	0.9985	0.9955	0.9954	0.9595	0.9895
	Gradient Boosting	0.9988	0.9989	0.9980	0.9961	0.9677	0.9919
	XGBoost	0.9989	0.9984	0.9971	0.9958	0.9238	0.9828
	Neural Network	0.9844	0.9811	0.9586	0.9394	0.7994	0.9326
Far Training Cluster 2	Average Method	0.8562	0.8582	0.8478	0.8595	0.8396	0.8523
	LASSO	0.9118	0.9110	0.9176	0.9117	0.5516	0.8407
	Ridge	0.8954	0.9630	0.9007	0.9002	0.6041	0.8388
	KNN	0.9166	0.9067	0.8840	0.9134	0.8348	0.8911
	SVM	0.9331	0.9065	0.9115	0.9093	0.8931	0.9107
	Random Forest	0.9833	0.9812	0.9714	0.9668	0.9466	0.9699
	Gradient Boosting	0.9858	0.9835	0.9743	0.9642	0.9525	0.9721
	XGBoost	0.9854	0.9826	0.9769	0.9554	0.9374	0.9675
	Neural Network	0.9833	0.9812	0.9714	0.9668	0.9466	0.9699

Table S6: MSE of the Average Method and Proposed ML Model Using Virtual Datasets of size 100

Training Cluster	Predictive Model	Sampling Time of Plasma					
		5 min	15 min	30 min	50 min	100 min	Overall
Near Training Cluster 3	Average Method	0.0066	0.0089	0.0427	0.1958	1.1219	0.2752
	Random Forest	0.0017	0.0013	0.0011	0.0023	0.0418	0.0096
	Gradient Boosting	0.0015	0.0011	0.0010	0.0013	0.0164	0.0043
	XGBoost	0.0025	0.0011	0.0014	0.0020	0.0240	0.0062
Far Training Cluster 2	Average Method	0.0486	0.0593	0.0978	0.2536	1.1704	0.3259
	Random Forest	0.0172	0.0159	0.0102	0.0116	0.2512	0.0612
	Gradient Boosting	0.0172	0.0243	0.0087	0.0089	0.2162	0.0551
	XGBoost	0.0157	0.00258	0.0085	0.0163	0.1804	0.0495

Table S7: R^2 values of the Average Method and Proposed ML Model Using Virtual Datasets with a sample size of 100

Training Cluster	Predictive Model	Sampling Time of Plasma					
		5 min	15 min	30 min	50 min	100 min	Overall
Near Training Cluster 3	Average Method	-1.0586	-1.6123	-0.0425	0.0000	-0.0104	0.8715
	Random Forest	0.4651	0.6280	0.9729	0.9882	0.9623	0.9955
	Gradient Boosting	0.5295	0.6707	0.9764	0.9935	0.9852	0.9980
	XGBoost	0.2106	0.6633	0.9653	0.9898	0.9784	0.9971
Far Training Cluster 2	Average Method	-14.2426	-16.3803	-1.3907	-0.2950	-0.0540	0.8478
	Random Forest	-4.4030	-3.6533	0.7503	0.9408	0.7738	0.9714
	Gradient Boosting	-4.3868	-6.1279	0.7869	0.9544	0.8053	0.9743
	XGBoost	-3.9226	-6.8420	0.7919	0.9170	0.8376	0.9769

Table S8: MSE of PPK and Proposed ML Model Using Real Datasets for Each Specified Time Point and Overall

Predictive Model	Sampling Time of Plasma					
	5 min	15 min	30 min	50 min	100 min	Overall
PPK model	0.0186	0.0374	0.0154	0.0479	0.1349	0.0508
Random Forest	0.0068	0.0307	0.0574	0.0792	0.0283	0.0405
(S.E.)	(0.0012)	(0.0188)	(0.0224)	(0.0297)	(0.0046)	(0.0132)
Gradient Boosting	0.0077	0.0288	0.442	0.0892	0.0329	0.0405
(S.E.)	(0.0004)	(0.0187)	(0.0247)	(0.0473)	(0.0070)	(0.0174)
XGBoost	0.0074	0.0324	0.0496	0.0761	0.0336	0.0402
(S.E.)	(0.0018)	(0.0116)	(0.0248)	(0.0290)	(0.0054)	(0.0129)

Table S9: R^2 values of PPK and Proposed ML Models Using Real Datasets for Each Specified Time Point and Overall

Predictive Model	Sampling Time of Plasma					
	5 min	15 min	30 min	50 min	100 min	Overall
PPK model	0.5618	0.8530	0.9364	0.7979	0.2928	0.9573
Random Forest	0.8248	0.7189	0.6570	0.6157	0.3889	0.9606
(S.E.)	(0.0315)	(0.1801)	(0.1424)	(0.1546)	(0.0332)	(0.0117)
Gradient Boosting	0.8234	0.7233	0.6979	0.6260	0.3017	0.9602
(S.E.)	(0.0317)	(0.1810)	(0.1567)	(0.1571)	(0.0867)	(0.0163)
XGBoost	0.7925	0.6404	0.6451	0.6057	0.3035	0.9618
(S.E.)	(0.0362)	(0.1647)	(0.1502)	(0.1592)	(0.1097)	(0.0107)

Table S10: MSE of PPK and Proposed ML Models Using Real Datasets for Each Fold of Time Points during the Infusion Period and Overall

Predictive Model	Sampling Time of Plasma					
	TINFCAT×0.5	TINFCAT×1.5	TINFCAT×3	TINFCAT×5	TINFCAT×10	Overall
PPK model	0.0111	0.0641	0.0149	0.0157	0.1428	0.0491
Random Forest	0.0120	0.0308	0.0573	0.0425	0.0696	0.0422
(S.E.)	(0.0025)	(0.0078)	(0.0180)	(0.0166)	(0.0177)	(0.0113)
Gradient Boosting	0.0105	0.0289	0.0452	0.0574	0.0658	0.0415
(S.E.)	(0.0022)	(0.0112)	(0.0188)	(0.0249)	(0.0242)	(0.0152)
XGBoost	0.0124	0.0267	0.0404	0.0513	0.0618	0.0384
(S.E.)	(0.0020)	(0.0073)	(0.0130)	(0.0163)	(0.0186)	(0.0095)

Table S11: R^2 value of PPK and Proposed ML Models Using Real Datasets for Each Fold of Time Points during the Infusion Period and Overall

Predictive Model	Sampling Time of Plasma					
	TINFCAT×0.5	TINFCAT×1.5	TINFCAT×3	TINFCAT×5	TINFCAT×10	Overall
PPK model	0.7719	0.7666	0.9022	0.9160	0.0635	0.9531
Random Forest (S.E.)	0.6741 (0.0951)	0.5887 (0.0961)	0.6092 (0.0584)	0.7666 (0.0520)	0.1528 (0.0814)	0.9569 (0.0105)
Gradient Boosting (S.E.)	0.7077 (0.1127)	0.6448 (0.1320)	0.6792 (0.0621)	0.7158 (0.0810)	0.2107 (0.0808)	0.9583 (0.0144)
XGBoost (S.E.)	0.6483 (0.0863)	0.6130 (0.0952)	0.6893 (0.0560)	0.7412 (0.0639)	0.1475 (0.0647)	0.9607 (0.0093)

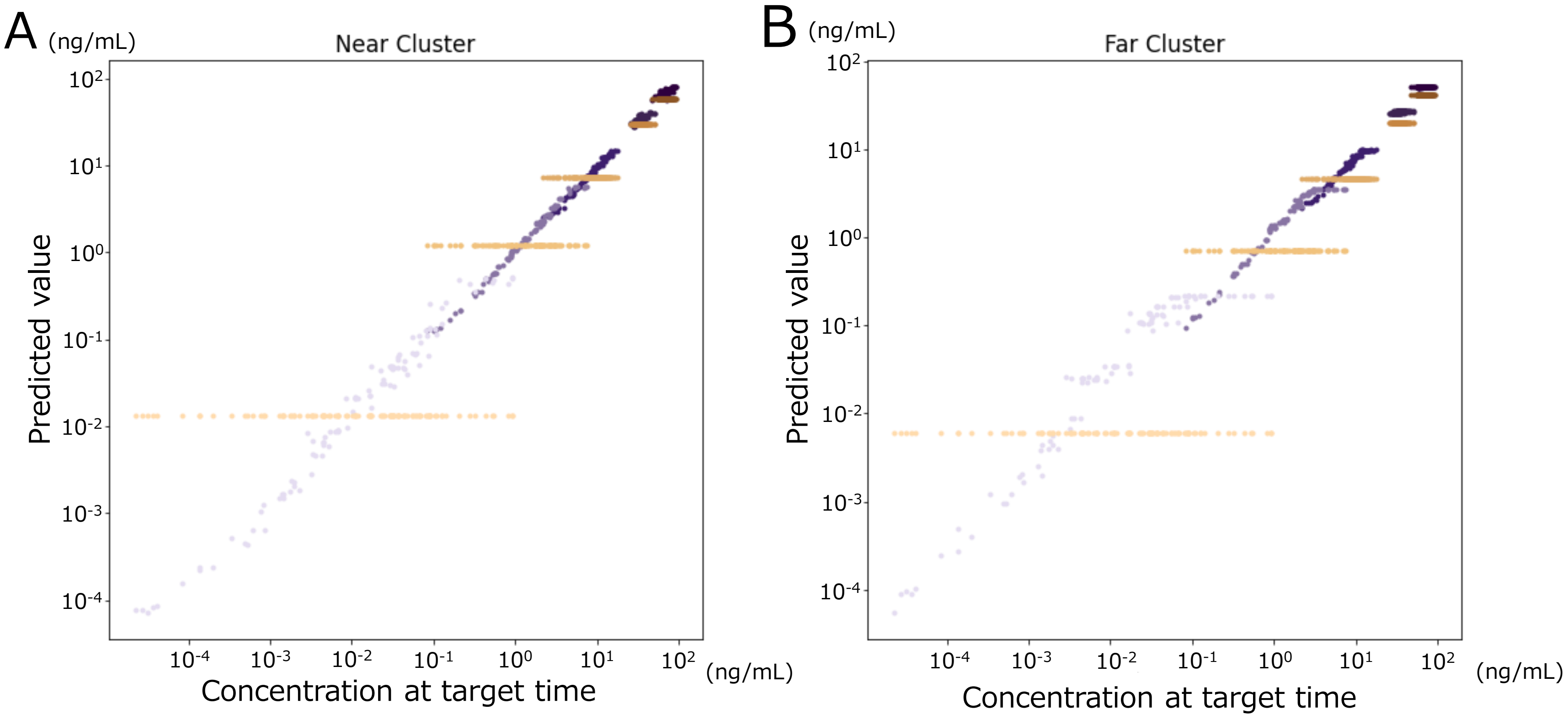

Figure S1: Comparison of predictivity at different Cp values using 100 training subjects from the near (A) and far (B) clusters
